# Supplementary material for: IKBKB reduces huntingtin aggregation by phosphorylating serine 13 via a non-canonical IKK pathway
Source: Life Sci Alliance. 2023 Aug 8;6(10):e202302006. doi: 10.26508/lsa.202302006 (PMC10410066; doi:10.26508/lsa.202302006)
Supplement: Supplementary file 1 [file LSA-2023-02006_TableS1.docx]

**Table. S1**

| **Construct name** | **DNA sequence** | **Protein sequence** |
| --- | --- | --- |
| exon 1 HTT 1-90,  based on Q23 numbering | ATGGCGACCCTGGAAAAGCTGATGAAGGCCTTCGAGTCCCTCAAGTCCTTCCAACAGCAGCAGCAGCAGCAGCAGCAGCAGCAGCAGCAGCAGCAGCAGCAGCAGCAGCAGCAGCAGCAGCCGCCACCGCCGCCGCCGCCGCCGCCGCCTCCTCAGCTTCCTCAGCCGCCGCCGCAGGCACAGCCGCTGCTGCCTCAGCCGCAGCCGCCCCCGCCGCCGCCCCCGCCGCCACCCGGCCCGGCTGTGGCTGAGGAGCCGCTGCACCGACCA | MATLEKLMKAFESLKSFQQQQQQQQQQQQQQQQQQQQQQQPPPPPPPPPPPQLPQPPPQAQPLLPQPQPPPPPPPPPPGPAVAEEPLHRP |
